# What and how should we measure in paediatric oncology FDG-PET/CT? Comparison of commonly used SUV metrics for differentiation between paediatric tumours

**DOI:** 10.1186/s13550-019-0577-7

**Published:** 2019-12-23

**Authors:** Janusch Blautzik, Leonie Grelich, Nicolai Schramm, Rebecca Henkel, Peter Bartenstein, Thomas Pfluger

**Affiliations:** 1 Department of Nuclear Medicine, University Hospital, LMU Munich, Munich, Germany; 2 Department of Radiology, University Hospital, LMU Munich, Munich, Germany; 3 Institute for Radiology and Nuclear Medicine, Hirslanden Klinik St. Anna, Lucerne, Switzerland; 4 Department of Nuclear Medicine, University Hospital, Würzburg, Germany

**Keywords:** FDG PET/CT, SUV, Paediatric oncology, Lymphoma, Sarcoma

## Abstract

**Background:**

In clinical routine, SUV_max_ and SUV_peak_ are most often used to determine the glucose metabolism in tumours by ^18^F-FDG PET/CT. Both metrics can be further normalised to SUVs in reference regions resulting in a SUV ratio (SUV_ratio_). The aim of the study was to directly compare several widely used SUVs/SUV_ratios_ with regard to differentiation between common tumours in paediatric patients; a special focus was put on characteristics of reference region SUVs.

**Methods:**

The final study population consisted of 61 children and adolescents with diagnoses of non-Hodgkin lymphoma (NHL, *n* = 25), Hodgkin lymphoma (HL, *n* = 14), and sarcoma (*n* = 22). SUV metrics included SUV_max_ and SUV_peak_ as well as both parameters normalised to liver and mediastinal blood pool, respectively, yielding the SUV_ratios_ SUV_max/liver_, SUV_max/mediastinum_, SUV_peak/liver_, and SUV_peak/mediastinum_.

**Results:**

The metrics SUV_max_, SUV_peak_, SUV_max/liver_, and SUV_peak/liver_ all proved to be sensitive for tumour differentiation (*p* ≤ 0.008); in contrast, SUV_max/mediastinum_ and SUV_peak/mediastinum_ revealed to be non-sensitive approaches. Correlation analyses showed inverse associations between reference region SUVs and SUV_ratios_ (*p* < 0.05). Multiple regression analyses demonstrated significant effects of factors as bodyweight and uptake time on reference region SUVs (*p* < 0.01), and thus indirectly on the corresponding SUV_ratios_.

**Conclusions:**

In the paediatric population, the ability to differentiate between common tumours remarkably varies between SUV metrics. When using SUV_ratios_, the choice of reference region is crucial. Factors potentially influencing reference region SUVs (and thus SUV_ratios_) should be taken into account in order to avoid erroneous conclusions. When not possible, SUV_max_ and SUV_peak_ represent less complex, more robust alternatives.

## Background

Children and adolescents with malignant solid tumours and lymphoma have in general a good prognosis with five-year survival rates exceeding 70% [1]. A fundamental requirement for the successful management of these tumours is the use of appropriate imaging methods for accurate disease detection and characterization. In this context, due to the increased glucose uptake and glycolysis of most cancer cells, metabolic imaging on a combined positron emission tomography/computed tomography (PET/CT) scanner with the radioactive glucose analogue 2-deoxy-2-(^18^F)fluoro-D-glucose (^18^F-FDG) is recognised as highly sensitive and specific. For instance, for detection of paediatric Hodgkin lymphoma (HL) and non-Hodgkin lymphoma (NHL), sensitivity and specificity has been reported to be as high as 95.9% and 99.7%, respectively, in a lesion-based regional analysis [2]; in case of paediatric sarcoma, sensitivities and specificities for staging of all lesions have been reported to be 81.8% and 97.5% for bone tumours [3], and 86% and 80% for soft tissue tumours [4].

Different quantitation methods have been proposed to determine the glucose metabolism within a tumour lesion by ^18^F-FDG PET/CT, each with its specific advantages and disadvantages [5]. For clinical routine and for scientific purposes, the concept of standardized uptake value (SUV) is generally accepted and most often used as SUV quantification is easy to perform and the correlation with more complex approaches is usually good [6–8]. For SUV determination, the tissue concentration of the tracer within a specific region-of-interest (ROI) as measured by the PET scanner is divided by the activity injected and normalised to body mass, lean body mass, or body surface area [5, 9]. Several approaches exist for ROI selection including fixed-dimension regions centered over the part of the tumour with the highest uptake (e.g., a sphere of 12 × 12 mm) resulting in a metric commonly called SUV_peak_ or simply determining the single hottest voxel within the tumour, the so-called SUV_max_ [5]. Moreover, the different semi-quantitative SUV measures can be further normalised to SUVs in unaffected regions as blood pool or normal liver in order to reduce variation and thus increase standardization resulting in a SUV ratio (SUV_ratio_) [10].

The ability to reproducibly quantify tumour metabolism has made SUV measures a powerful tool in ^18^F-FDG PET/CT imaging in oncology. Beside their use in the diagnosis of malignancy, SUV measures may also provide information about different tumor profiles with different aggressiveness and consequently prognosis given that the degree of FDG uptake is usually associated with tumour histology [11]. For instance, in the special case of paediatric oncology, SUV_max_ has been demonstrated, amongst others, to be predictive of outcomes in Ewing sarcoma (ES) [12, 13], rhabdomyosarcoma (RMS) [14], and HL [15], and SUV_peak_ was reported to be a useful prognostic biomarker for osteosarcoma (OS) [16]. Furthermore, SUV measures may also be used for therapy monitoring of antineoplastic treatments. In this regard, the principle behind the SUV_ratio_ concept, which is used in several approaches, e.g., PERCIST [5], the visual Deauville criteria [17] or a quantitative extension of the Deauville scale referred to as qPET, has been proposed for the assessment of tumour response to therapy by ^18^F-FDG PET/CT in paediatric HL patients [18].

Despite the wide use of various SUV metrics there exist to our knowledge no studies directly comparing these measures in paediatric oncology. In order to contribute to this topic, we retrospectively analysed ^18^F-FDG PET/CT data of children and adolescents with the diagnosis of lymphoma and sarcoma, which together account for approximately 25% of all malignancies in the paediatric population [19–21]. In particular, we tested the ability of SUV_max_, SUV_peak_, as well as of both parameters normalised to the liver and mediastinal blood pool, respectively, to differentiate between these malignancies at initial staging and, in that context, also evaluated these measures’ characteristics in more detail. This involved taking a closer look at possible associations between reference regions SUVs and SUV_ratios_ as well as at the potential impact of factors such as bodyweight or uptake time (i.e., the time between PET tracer injection and beginning of imaging) on reference region SUVs and thus on SUV_ratios_.

A broad understanding of SUV measures with their specific characteristics and potential pitfalls in paediatric patients, which according to common view are not little adults, is in general mandatory for the accurate interpretation of oncologic PET/CT studies in this population with regard to detection and characterization of the tumour in clinical routine as well as prognosis estimation in clinical routine and scientific investigations, respectively. This is also true for interpreting the response to treatment.

## Methods

### Subjects

All procedures performed in the study involving human participants were in accordance with the ethical standards of the Institutional Review Board and with the 1964 Helsinki Declaration and its later amendments. The study was approved by the local ethics committee.

The electronic database of all PET/CT scans performed between the years 2011 and 2016 at the authors’ University Hospital was searched to identify children and adolescents with the initial diagnosis of NHL, HL, and sarcoma who underwent imaging for initial staging purposes. The search yielded imaging data of 75 patients: NHL, *n* = 30; HL, *n* = 20; RMS, *n* = 8; OS, *n* = 8; and ES, *n* = 9. Data were excluded from the analysis in the following cases: multifocal liver involvement (2 NHL; no adequate SUV measurement in healthy hepatic reference tissue possible); lacking of sufficient histological data (2 HL); recurrent disease with primary diagnosis not done at our institution (3 NHL, 4 HL, 1 OS); imaging performed after beginning of chemotherapy (1 ES) or after tumor resection (1 ES). Thus, the final study population consisted of 61 children and adolescents (age range 1–17 years) with the diagnosis of NHL (*n* = 25), HL (*n* = 14), RMS (*n* = 8), OS (*n* = 7), and ES (*n* = 7).

### ^18^F-FDG PET/CT acquisition

All patients fasted for at least 6 hours and had serum glucose levels < 120 mg/dl. Furosemide and butylscopolamine were administered prior to ^18^F-FDG for diuresis and to decrease bowel activity. Dosage was 10 mg for each drug for children < 10 years and 20 mg for older children and adolescents. ^18^F-FDG was given as an intravenous injection adapted to the patient’s weight according to the EANM dosage card. Imaging covered the whole body with the patient in the supine position. The need for sedation and anaesthesia was assessed individually and the appropriate procedure was provided by a dedicated anaesthetics team.

All studies have been performed on one and the same GE Discovery 690 PET/CT system (General Electric, USA) combining a lutetium-yttrium-orthosilicate block detector designed PET and a 64-slice CT.

A diagnostic CT with intravenous contrast agent was performed with the following parameters adapted to patient´s age: < 5 years: 80 kV, 40 mA; 5–12 years: 100 kV, 40–60 mA; 17–18 years: 100 kV, 60 mA. For patients with recent MRI examinations, an unenhanced low dose CT protocol with 100 kV and 10 mA was applied.

PET scans were acquired in a 3D mode (144 × 144 matrix) in a caudocranial direction and corrected for decay and scatter. PET data were reconstructed iteratively by using the CT images for attenuation correction. Combined transaxial images of ^18^F-FDG PET and CT were reconstructed to a resolution of 128 × 128 and a thickness of 2.5 mm resulting in a voxel size of approx. 75 mm^3^.

### SUV quantitation methods

Parameters for SUV calculation included the injected dose of ^18^F-FDG and the patient’s body surface area; the latter was calculated based on patient’s height and bodyweight. SUV metrics included SUV_max_ and SUV_peak_ as well as both parameters normalised to liver and mediastinal blood pool, respectively, yielding the SUV_ratios_ SUV_max/liver_, SUV_max/mediastinum_, SUV_peak/liver_, and SUV_peak/mediastinum_. For both, liver and mediastinal blood pool, a mean SUV of all voxels located in a representative target volume was calculated, resulting in the metrics SUV_liver_ and SUV_mediastinum_. For the hepatic target volume, a spherical ROI with a diameter of 3 cm was placed to the right lobe showing homogeneous FDG uptake [5]; in case of mediastinal blood pool, a spherical ROI of 2 cm in diameter was placed to the right ventricle of the heart. SUV metrics were determined for the patient’s most active tumour lesion (which was chosen after measuring all lesions within the patient).

Analyses were performed on a workstation using the software HERMES Gold, version 4.16 (Hermes Medical Solutions AB, Sweden)*.*

### Statistical analyses

Preparatory analyses showed that for several SUV metrics the assumption of homogeneity of variance across tumour groups was violated. Therefore, the more robust Welch ANOVA was calculated separately for SUV_max_, SUV_peak_, SUV_max/liver_, SUV_max/mediastinum_ , SUV_peak/liver_, and SUV_peak/mediastinum_. To protect from type 1 error, a Bonferroni correction was performed accepting Welch ANOVA results as statistically significant at *p* ≤ 0.008. Post hoc analyses were conducted using the Games-Howell test. As preparatory analyses did not show any significant differences within the sarcoma group (*p* > 0.05), patients with RMS, OS, and ES were combined resulting in total in three tumour entity groups (i.e., NHL, HL, and sarcoma).

For the same reason as mentioned above, Welch ANOVAs with Games-Howell tests were used to test for between-group differences in age, bodyweight, injected dose of ^18^F-FDG, and uptake time; in this case, Welch ANOVA results were considered statistically significant at Bonferroni-adjusted *p* ≤ 0.0125. Potential differences in gender distribution between groups were tested with the chi-square test.

A one-way MANOVA was conducted to test for differences in reference region SUVs among tumour entity groups; significant ANOVAs were followed up with Tukey’s HSD post hoc test.

Pearson correlation coefficients were calculated in order to study the associations between SUV metrics in general and the impact of reference region SUVs on tumour lesion SUV_ratios_ in particular.

To extract factors that may influence reference regions SUVs, multiple regressions were run including the factors gender, age, weight, dose of ^18^F-FDG, and uptake time.

Statistical data was computed with SPSS, version 23.0 (IBM, USA).

## Results

Table 1 summarizes patients’ data regarding age, bodyweight, injected dose of ^18^F-FDG, and uptake time. No statistically significant differences (*p* > 0.125 each) between patient groups were present for age (*F*(2, 35.29) = 4.797), weight (*F*(2, 35.03) = 4.462), and dose of ^18^F-FDG (*F*(2, 34.61) = 1.990). Uptake time, defined as the time between PET tracer injection and beginning of imaging, differed significantly between groups (*F*(2, 34.84) = 8.381, *p* = 0.001); it was shorter in HL patients than in those with NHL (− 9.18 min, *p* = 0.021) and sarcoma (− 18.79 min, *p* = 0.004). No significant gender differences were present across groups (*χ*^*2*^ = 0.894, *p* > 0.05).

**Table 1:** Subject’s age, bodyweight, injected dose of ^18^F-FDG, and uptake time: means ± standard deviation

	All patients	NHL	HL	Sarcoma
Age, years	10.33 ± 4.97 [1–17]	9.8 ± 5.0	13.1 ± 3.7	8.7 ± 5.2
Bodyweight, kg	38.50 ± 20.69 [7.0–89.5]	36.3 ± 20.3	49.9 ± 15.4	32.2 ± 21.9
^18^F-FDG dose, MBq	134.97 ± 53.65 [41–246]	129.8 ± 51.8	155.1 ± 40.9	124.3 ± 62.0
Uptake time, min	70.14 ± 16.94 [46–126]	69.0 ± 12.7	59.9 ± 7.4	78.7 ± 21.7

*NHL* non-Hodgkin lymphoma, *HL* Hodgkin lymphoma. Uptake time: time between PET tracer injection and the beginning of imaging in minutes

Most active tumour lesions (i.e. those used for the analysis) included lymph nodes/lymphatic tissue in the neck (*n* = 2), mediastinum (*n* = 9), retroperitoneal (*n* = 6) and intraperitoneal (*n* = 5) space, and pelvis (*n* = 3) for NHL; lymph nodes in cervical/supraclavicular (*n* = 6), axillary (*n* = 2), and mediastinal (*n* = 6) regions in case of HL; head and neck region (*n* = 4), retroperitoneal space (*n* = 2), upper and lower extremities (1 each) for RMS; humerus (*n* = 1), femur (*n* = 3), tibia (*n* = 2), and fibula (*n* = 1) for OS; and humerus (*n* = 1), radius (*n* = 1), ribs (*n* = 1), pelvis (*n* = 2), femur (*n* = 1), and tibia (*n* = 1) in case of ES.

**Fig. 1:**
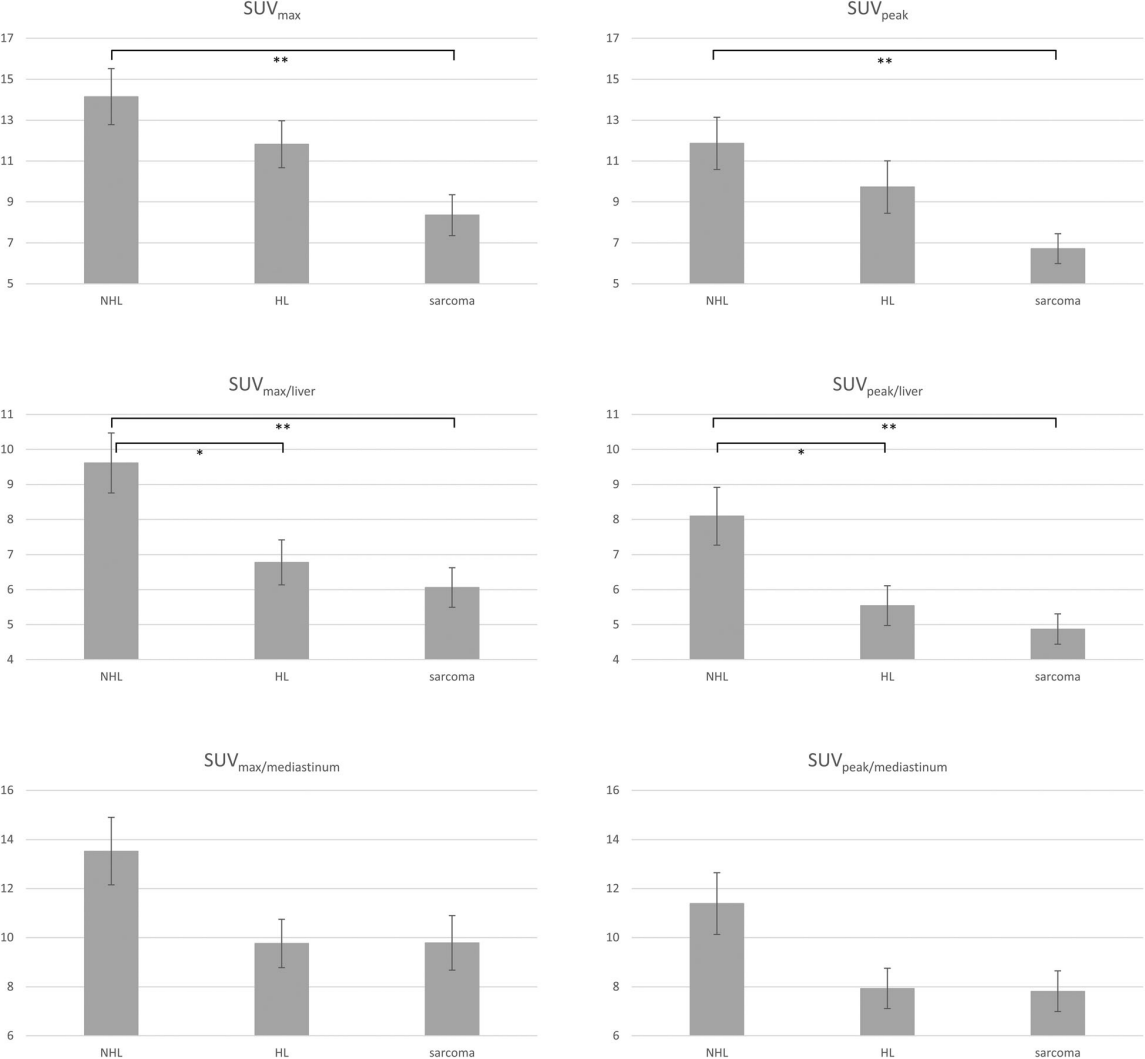
Mean SUVs/SUV_ratios_ ± standard error across tumor entities. NHL, non-Hodgkin lymphoma. HL, Hodgkin lymphoma. *p < 0.05; **p < 0.01

Mean tumour lesion SUV metrics for each tumour entity are summarized in Table 2; Fig. 1 illustrates the results graphically:

**Table 2:** SUV metrics: means ± standard deviation

	NHL	HL	Sarcoma
SUV_max_	14.15 ± 6.85 [11.32–16.98]	11.82 ± 4.30 [9.33–14.30]	8.36 ± 4.45 [6.27–10.44]
SUV_peak_	11.86 ± 6.42 [9.22–14.51]	9.72 ± 3.84 [7.51–11.94]	6.72 ± 3.27 [5.19–8.25]
SUV_max/liver_	9.61 ± 4.29 [7.84–11.38]	6.78 ± 2.40 [5.39–8.17]	6.06 ± 2.53 [4.88–7.24]
SUV_max/mediastinum_	13.52 ± 6.86 [10.69–16.36]	9.76 ± 3.70 [7.62–11.90]	9.78 ± 4.98 [7.45–12.11]
SUV_peak/liver_	8.09 ± 4.11 [6.40–9.79]	5.54 ± 2.13 [4.32–6.77]	4.87 ± 1.94 [3.96–5.78]
SUV_peak/mediastinum_	11.39 ± 6.31 [8.78–13.99]	7.93 ± 3.06 [6.17–9.70]	7.81 ± 3.69 [6.09–9.54]

*NHL* non-Hodgkin lymphoma, *HL* Hodgkin lymphoma. In brackets: 95% confidence interval

Mean SUVs/SUV_ratios_ differed statistically significantly between tumour entities for SUV_max_, SUV_peak_, SUV_max/liver_, and SUV_peak/liver_, but not for SUV_max/mediastinum_ and SUV_peak/mediastinum_. Table 3 shows details of the Welch ANOVA:

**Table 3:** Results of the Welch ANOVA

SUV metric	Welch‘s *F*	*p* value
SUV_max_	*F*(2, 35.18) = 6.279	0.005
SUV_peak_	*F*(2, 33.64) = 6.975	0.003
SUV_max/liver_	*F*(2, 35.51) = 5.976	0.006
SUV_max/mediastinum_	*F*(2, 36.76) = 2.847	0.071
SUV_peak/liver_	*F*(2, 34.34) = 5.882	0.006
SUV_peak/mediastinum_	*F*(2, 36.55) = 3.144	0.055

Uptake was found to be significantly higher in NHL lesions than in sarcoma (*p* = 0.004 each) for SUV_max_ (+ 5.79), SUV_peak_ (+ 5.15), SUV_max/liver_ (+ 3.55), and SUV_peak/liver_ (+ 3.22). In addition, significantly higher values were present in NHL compared to HL for SUV_max/liver_ (+ 2.83, *p* = 0.031) and SUV_peak/liver_ (+ 2.55, *p* = 0.039).

After excluding subjects with multifocal liver involvement, no conditions were identifiable in the study population based on all available information including past medical history and imaging data that could have negatively affected SUV measurements within liver or mediastinal blood pool (e.g. excretory dysfunction due to infiltration/compression of the nephrourinary system with consecutively reduced tracer clearance from blood and body tissues). Table 4 shows the results for mean liver and mediastinal blood pool SUVs for each patient group.

**Table 4:** Reference region SUVs: means ± standard deviation

Reference region SUV	NHL	HL	Sarcoma
SUV_liver_	1.58 ± 0.64	1.83 ± 0.49	1.41 ± 0.40
SUV_mediastinum_	1.13 ± 0.37	1.31 ± 0.42	0.92 ± 0.30

*NHL* non-Hodgkin lymphoma; *HL* Hodgkin lymphoma

The MANOVA revealed a significant multivariate main effect for tumour group (*F*(4,110) = 2.63, *p* = 0.038, Wilk’s Λ = 0.833, partial η^2^ = 0.09). Significant univariate main effects were obtained for SUV_mediastinum_ (*F*(2,56) = 4.85, *p* = 0.011, partial η^2^ = 0.15) but not for SUV_liver_ (*F*(2,56) = 2.52, *p* > 0.05, partial η^2^ = 0.08). Mean mediastinal blood pool SUV was significantly higher in patients with HL than in those with sarcoma (+ 0.39, *p* = 0.009). No differences were identifiable between patients with NHL and HL, and NHL and sarcoma (*p* > 0.05).

Pearson correlations indicating the associations between tumour SUVs/SUV_ratios_ and reference region SUVs are shown in Table 5. High correlations existed between SUV_max_ and SUV_peak_ as well as between SUV_ratios_. Correlations were usually lower between tumour SUVs and SUV_ratios_. SUV_max_ and SUV_peak_ were also associated with reference region SUVs. Accordingly, there was to some extent an inverse correlation between reference region SUVs and SUV_ratios_.

**Table 5:** Correlation matrix displaying Pearson correlations across tumour SUVs/SUV_ratios_ and reference region SUVs

	SUV_max_	SUV_peak_	SUV_liver_	SUV_mediastinum_	SUV_max/liver_	SUV_max/mediastinum_	SUV_peak/liver_
SUV_peak_	0.986***						
SUV_liver_	0.414**	0.399**					
SUV_mediastinum_	0.300*	0.299*	0.864***				
SUV_max/liver_	0.684***	0.689***	(0.295)*	(0.310)*			
SUV_max/mediastinum_	0.690***	0.672***	(0.212)	(0.393)**	0.930***		
SUV_peak/liver_	0.663***	0.696***	(0.282)*	(0.281)*	0.984***	0.890*	
SUV_peak/mediastinum_	0.692***	0.704***	(0.208)	(0.363)**	0.948***	0.981***	0.941***

*p < 0.05; **p < 0.01; ***p < 0.001. Numbers in parenthesis represent negative associations

Gender, age, weight, dose of ^18^F-FDG, and uptake time statistically significantly predicted SUV_liver_ (*F*(5,52) = 25.022, *p* < 0.0001, *R*^2^ = 0.706) and SUV_mediastinum_ (*F*(5,52) = 26.348, *p* < 0.0001, *R*^2^ = 0.717). In case of SUV_liver_, female gender (β = 0.218, *p* = 0.008) and weight (β = 0.687, *p* < 0.001) added significantly to the prediction; in case of SUV_mediastinum_, these were weight (β = 0.681, *p* < 0.001) and uptake time (β = − 0.281, *p* < 0.001).

## Discussion

Our retrospective data suggests that the ability to differentiate between common tumours in paediatric patients remarkably varies between SUV metrics. It also shows that there is an inverse association between reference region SUVs and the corresponding SUV_ratios_, especially when SUV_mediastinum_ is used, indicating that uptake behaviour of reference regions may substantially impact SUV_ratios_.

Liver and mediastinal blood pool SUVs were both predicted by the patient’s weight; in addition, SUV_liver_ was also affected by gender, and SUV_mediastinum_ by uptake time. To the best of our knowledge, the association between SUVs in liver/mediastinal blood pool and these factors has not yet been investigated in children and adolescents. However, prior research on adult patients have repeatedly shown that bodyweight affects SUVs in both reference regions, e.g., the work by Malladi et al. [22] or a study by Groheux et al., who concentrated on the effect of weight on liver only [23]. Malladi et al. also found effects of gender on liver and mediastinal blood pool SUV [22], a finding that in our work on paediatric patients could be replicated for liver only. Regarding the negative impact of uptake time on SUV_mediastinum_ our finding is in line with data reported for adults [22, 24], presumably due to continuing renal excretion of the tracer over time.

Uptake time substantially varied across patient groups. The reason for this unintended result was a different need for sedation and anaesthesia between groups (data not shown here); the HL group involved more of the older children (13.1 ± 3.7 years), whereas the NHL (9.8 ± 5.0 years) and especially the sarcoma group (8.7 ± 5.2 years) included more of the very young patients where need for time-consuming sedation or anaesthesia—and thus probability of scan delays—was higher. Consecutively, uptake time was generally shorter in HL patients (59.9 ± 7.4 min) than in those with NHL (69.0 ± 12.7 min) and sarcoma (78.7 ± 21.7).

Given the considerably shorter uptake time in HL than in sarcoma patients combined with an inverse association between uptake time and mediastinal blood pool SUV, it is not surprising that SUV_mediastinum_ was significantly higher in HL patients than in those with sarcoma. Considering also a generally more pronounced impact of SUV_mediastinum_ on SUV_ratio_, we reason that the between-group differences in uptake time substantially biased both SUV_max/mediastinum_ and SUV_peak/mediastinum_ and thus abolished these metrics’ ability to differentiate between tumour entities. But we acknowledge that measuring the SUV_mean_ in a small volume as the right ventricle of a child may be difficult and thus prone to inaccurate measurements, which may have contributed to the low performance of these metrics.

Besides the obvious susceptibility of SUV_max/mediastinum_ and SUV_peak/mediastinum_ to variations in uptake time, use of liver as reference tissue may also be critical given the liver SUV’s association with bodyweight and gender; for this reason, the results found for SUV_max/liver_ and SUV_peak/liver_ should be interpreted cautiously as they may be biased by between-group differences in these factors, too (even though they were not found to be statistically significant in this study).

In general, our findings suggest that in children and adolescents validity of SUV_ratios_ is compromised in cases where the metric is based on reference regions highly sensitive to factors like gender, uptake time and/or bodyweight. Ignoring these factors may thus lead to erroneous study interpretations. This can be the case for SUV_ratio_ comparisons between different groups as well as within groups over time in scientific studies when substantial between-group or between-examination differences in these factors exist. In clinical routine, misinterpretations of longitudinal PET/CT studies may happen, which, in worst case, could have adverse effects on patients management, e.g. by negatively impacting the decision on follow-up examinations whether or not to continue a specific therapy regimen; but also at initial staging, the extent of the disease could be over- or underestimated in cases where SUVs in reference tissues are abnormally altered, thus potentially influencing treatment options or prognosis estimation. In the paediatric population, the role of bodyweight may be of particular importance given that relative differences in this factor can be substantial between groups (e.g. when comparing groups of different ages) but also longitudinally within a group or in an individual, as children grow and get heavier over time. It follows that choice of reference regions is crucial.

If it can be ensured that biasing factors are known and adequately controlled for, SUV_ratios_ may represent suitable SUV metrics. In all other cases, it seems reasonable that tumour lesion SUVs are preferred over SUV_ratios_ in paediatric oncology. Here, the eschewal of a reference tissue apparently makes the SUV_max_ or SUV_peak_ more robust as it decreases the degree of complexity. But it should be noted that both SUV metrics also have their disadvantages. Beside some technical issues [5], the concentration of ^18^F-FDG is known to gradually increase with time within glucose-avid tumour lesions [25], so the factor uptake time should also here be taken into account.

SUV_max_ and SUV_peak_ were highly correlated delivering similar results with regard to tumour differentiation, independent of their use solely or in combination with reference regions. This similarity is remarkable as the parameters rely on uptake information derived from substantially different volumes (75 mm^3^ vs. 1 cm^3^ in our study). In general, due to its small size, SUV_max_ is known to be adversely affected by noise and to be sensitive to respiratory motion artifacts that naturally occur during PET acquisition [26, 27]. SUV_peak_ on the other hand is less affected by these inherent disadvantages of SUV_max_ and thus preferred by some authors [5]; in contrast, however, SUV_peak_ is more prone to partial volume effects [5]. We think that the similarity of both values in this study is linked to the population examined. In comparison to adults, paediatric patients are smaller in total volume and so are the volumes of their malignancies (but also their motion range and thus potential motion artifacts). A SUV_max_ derived from a small volume will more likely capture a representative uptake value within a smaller paediatric tumour lesion and it should be less affected by motion artifacts than in adults. Partial volume effects associated with the SUV_peak_, on the other hand, could be more problematic in small paediatric tumours. In conclusion, it seems that in the special case of paediatric oncologic PET imaging, the (dis-)advantages of either SUV metric tend to balance each other, thus delivering similar results, at least with the specifications used in the current study.

However, it has to be noted that SUVs are in general imperfect parameters as they are altered by various factors that can result in considerable variations of accuracy and reproducibility. For instance, technical errors may derive from alterations in the calibration of the PET scanner and the dose calibrator or by paravenous tracer injection, effecting SUVs by ± 10% and up to 50%, respectively; biologic factors as patient motion may result in SUV alterations by up to 30%; finally, data acquisition and processing parameters may lead to further significant variability [28, 29]. Thus, the implementation of standardized scan and good quality control procedures as well as the consideration of sources of variability are mandatory for PET/CT analysis.

There are several limitations of the current study that must be taken into consideration. First, we evaluate the ability of different SUV metrics for differentiation between tumours by implicitly assuming that differences in glucose consumption between these entities exist. To our knowledge, studies illuminating this issue in paediatric patients have not yet been performed. Also, we combine different tumor subtypes into three tumor entity groups. This type of data reduction can be considered problematic as substantial differences in FDG uptake may also exist on the subtype level of a tumor entity.

Also, it has to be noted that more complex methods to determine a tumour’s metabolic characteristics such as metabolic tumour volume (MTV) and total lesion glycolysis (TLG) were not evaluated. These so-called second-order metrics, also based on the SUV concept, are very interesting as they—in contrast to first order metrics as SUV_max_ or SUV_peak_—try to capture the metabolic information from the entire tumour volume. And indeed, several studies report very promising results regarding the prognostic value of these parameters for various lymphoma types [30, 31] as well as for soft tissue and bone sarcoma [16, 32]. However, studies comparing the value of MTV and TLG with simple SUV_max_ and SUV_peak_ metrics as used in the current work do not show a clear advantage of the second-order methods in these tumour types [33–35]. Moreover, to the best of the authors’ knowledge quantitation of tumour metabolism with simple first-order SUV metrics is still most widely used in clinical routine as well as in the scientific world and thus a broader understanding of these measures is of great importance.

For the current work, we used CT scans with and without intravenous contrast agent for attenuation correction of PET data. SUV values calculated from contrast-enhanced CT’s will be slightly higher than SUV values calculated from non-contrast CT's; the difference is usually quite small [36], but may affect SUV based analyses as performed here.

Moreover, our analysis of factors potentially influencing FDG concentration within liver and mediastinal blood pool is not complete; e.g. a well-known factor potentially affecting uptake within these regions, the blood glucose concentration [37, 38], was not taken into consideration in the current study as results of glucose testing were not recorded in the majority of cases, thus no sufficient data was available for further evaluation. Also, the effects of furosemide and butylscopolamine could not be investigated. However, research on adults has demonstrated that furosemide effectively eliminates ^18^F-FDG activity from the lower urinary tract and thus improves the diagnostic accuracy in abdominopelvic malignancies [39]; interestingly, furosemide was shown to not substantially affect tracer distribution within the mediastinal blood pool and soft tissues, respectively [40]. Butylscopolamine, on the other hand, reduces artefacts coming from bowel peristalsis, thus also improving the accuracy of abdominal ^18^F-FDG PET reporting [41]. Given that all patients in our study were administered furosemide and butylscopolamine, diagnostic accuracy of the PET data and effects on SUV measurements (including reduction of activity spill-over to lesions adjacent to the urinary system or bowel) should be comparable; some variability, however, may come from different dosages based on patient age (10 mg for each drug for children < 10 years and 20 mg for older children and adolescents).

Finally, we use a retrospective study design and a small sample size that reduces the statistical power. Therefore, our results and the implications made should be replicated and verified in future studies using a prospective setting and larger patient numbers.

## Conclusions

Our work shows that the ability to differentiate between common tumours in paediatric patients substantially varies between SUV metrics. In the case of SUV_ratios_, the choice of reference region is crucial as reference region SUVs may be significantly biased by factors like gender, uptake time, and bodyweight. Thus, these factors should be adjusted for. If not possible, we recommend the use of SUV_max_ and SUV_peak_; these metrics represent less complex and more robust—while imperfect—alternatives. Moreover, in the special case of the paediatric population, it apparently does not matter if SUV_max_ or SUV_peak_ is used as both parameters deliver very similar results, likely due to overall smaller volumes in these patients compared to adults.

## Data Availability

The datasets used and analysed during the current study are available from the corresponding author on reasonable request.

## Abbreviations

^18^F-FDG: 2-deoxy-2-(^18^F)fluoro-D-glucose
ES: Ewing sarcoma
HL: Hodgkin lymphoma
NHL: non-Hodgkin lymphoma
OS: Osteosarcoma
PET/CT: Positron emission tomography/computed tomography
RMS: Rhabdomyosarcoma
ROI: Region-of-interest
SUV: Standardized uptake value
SUV_ratio_: Standardized uptake value ratio
